# Effect of different feeding strategies and dietary fiber levels on energy and protein retention in gestating sows

**DOI:** 10.1093/jas/skae092

**Published:** 2024-04-25

**Authors:** Sigrid J Wisbech, Tina S Nielsen, Knud E Bach Knudsen, Peter K Theil, Thomas S Bruun

**Affiliations:** Department of Animal and Veterinary Sciences, Aarhus University AU-Viborg, DK-8830 Tjele, Denmark; Department of Animal and Veterinary Sciences, Aarhus University AU-Viborg, DK-8830 Tjele, Denmark; Department of Animal and Veterinary Sciences, Aarhus University AU-Viborg, DK-8830 Tjele, Denmark; Department of Animal and Veterinary Sciences, Aarhus University AU-Viborg, DK-8830 Tjele, Denmark; SEGES Innovation, DK-8200 Aarhus N, Denmark

**Keywords:** backfat, body condition, body retention, de novo fat, feed efficiency, nitrogen utilization

## Abstract

The aim of the study was to investigate whether increased inclusion of sugar beet pulp (**SBP**) alters retention of fat, protein, and energy when backfat (**BF**) is restored in early- and mid-gestation. In total, 46 sows were fed one of four dietary treatments with increasing inclusion of SBP providing dietary fiber (**DF**) levels of 119, 152, 185, and 217 g/kg; sows were assigned to one of three feeding strategies (**FS**; high, medium, and low) depending on BF thickness at mating and again at day 30 for the following month. On days 0, 30, 60, and 108, body weight (**BW**) and BF thickness were measured and body pools of protein and fat were estimated using the deuterium oxide technique. On days 30 and 60, urine, feces, and blood samples were collected to quantify metabolites, energy, and nitrogen (**N**) balances. On days 15 and 45, heart rate was recorded to estimate heat energy. At farrowing, total born and weight of the litter were recorded. In early gestation, BW gain (*P* < 0.01) and body protein retention increased (*P* < 0.05) with increasing fiber inclusion, while body fat retention increased numerically by 59%. The increase in BF was greatest for sows fed the high FS, intermediate when fed the medium strategy, and negligible for sows fed the lowest FS (*P* < 0.001). Nitrogen intake, N loss in feces, and N balance increased linearly, whereas N loss in urine tended to decrease with increasing inclusion of fibers in early gestation. Concomitantly, fecal energy output and energy lost as methane increased linearly (*P* < 0.001), while energy output in urine declined linearly. Total metabolizable energy (**ME**) intake therefore increased from 36.5 MJ ME/d in the low fiber group to 38.5 MJ ME/d in the high fiber group (*P* < 0.01). Changing the ME towards more ketogenic energy was expected to favor fat retention rather than protein retention. However, due to increased intake of ME and increased N efficiency with increasing fiber inclusion, the sows gained more weight and protein with increasing fiber inclusion. In conclusion, increased feed intake improved both fat and protein retention, whereas increased DF intake increased protein retention.

## Teaser

Increased dietary inclusion of sugar beet pulp fed to sows in early gestation numerically increased fat retention and unintendedly protein retention.

## Introduction

A challenge in practical pig production is to control the body condition of sows throughout the reproductive cycle. Hyperprolific sows mobilize substantial amounts of body fat for demanding milk production (Krogh et al., 2017; Strathe et al., 2017), and it is essential to restore lost body fat and protein during the following gestation period.

Fat deposition occurs when animals ingest excess energy, but the genetic selection has favored protein accretion over fat accretion in modern pig breeds. Energy in a normal diet for gestating sows mainly derives from glucose from digested starch, which can be used for either oxidation or de novo fat synthesis. Amino acids may be used as energy also, but are prioritized for protein accretion, and this process is also fueled by glucose oxidation. Fibers are fermented in the hindgut, and energy is absorbed as short-chain fatty acids (**SCFA**), mainly acetate, propionate, and butyrate. Ketogenic substrates, acetate, and butyrate, are more suitable for de novo fat synthesis than glucose because two of six carbons are lost as CO_2_ when glucose is the precursor for de novo fat synthesis (Theil et al., 2020), whereas all carbons in acetate and butyrate can be utilized. The Danish energy evaluation system for pigs focuses on the potential energy value of feedstuffs when nutrients are completely oxidized (Kil et al., 2013). However, it does not take into account that within the body carbon is utilized much more efficiently (100% for SCFA vs. 67% for glucose) when absorbed SCFA are used as precursors for de novo fat synthesized rather than absorbed glucose.

Dietary fibers (**DF**) have a wide range of beneficial effects. For instance, more DF decrease the diurnal variation in energy being absorbed to portal vein, because the uptake of SCFA is fairly constant during the post-prandial period, whereas the absorption of glucose peaks approximately 60 min after feeding and then decline (Serena et al., 2009). DF are also known to reduce physical activity (Rijnen et al., 2001), aggression, stress, and negative stereotypic behavior associated to gestating sows (Priester et al., 2020), which especially in early gestation are risk factors for early embryonic mortality (Peltoniemi et al., 2021). Housing animals individually or in smaller uniform groups limit these risk factors (Peltoniemi et al., 2016), and provide an opportunity to feed strategically to reach a certain target of backfat (**BF**). In this context, it is important to understand the influence of feed level and composition on muscle and fat retention of gestating sows when taking sow productivity, feed efficiency, health, and welfare into account.

It is hypothesized that increasing the intake of fiber obtained through diet composition and feeding strategy would improve energy utilization of sows when restoring BF, due to improved carbon (energy) utilization, reduced physical activity, and reduced heat loss.

## Materials and Methods

The present experiment complied with the Danish ministry of Justice Law number 382 (June 10, 1987), Act number 726 (September 9, 1993, as amended by Act number 1081 on December 20, 1995), concerning experiments with the care of animals. The Danish Animal Experimentation Inspectorate approved the study protocol (License number: 2018-15-0201-01484).

### Animals and housing

A total of 46 multiparous hybrid sows (DanBred landrace × DanBred Yorkshire) were stratified for BF thickness and body weight (**BW**) at weaning and allocated to four different treatments. The experiment was carried out in two blocks of 25 and 21 sows.

Animals were housed individually until day 60, in crates (65 cm × 245 cm) with partly slatted floor. From day 60, sows were group-housed until entering the farrowing unit around day 108. When group-housed, single crate with feed trough was used voluntarily by the sows, feeding was supervised with the possibility to lock the crates, to ensure enough time for each sow to eat the meal.

The temperature was kept constant at 18 °C and light was turned on 18 h each day. Ad libitum water intake was not monitored. The trial was conducted at Aarhus University, Foulum.

The sows were inseminated with mixed Duroc (DanBred Duroc, Hatting Agro, Horsens, Denmark) semen.

### Experimental design, dietary treatments, and feeding

Two diets (low and high fiber) were formulated based on wheat, barley, and soybean meal, and SBP partly replaced wheat in the high-fiber diet (Table 1). Both diets were formulated to contain the required amounts of nutrients per unit of net energy according to Danish recommendations for gestating sows (Tybirk et al., 2020). These two diets were produced by Vestjyllands Andel (Ringkøbing, Denmark), and at the experimental facility at Aarhus University, two additional treatments (33 % low/67% high and 67% high/33% low DF diets) were mixed before each feeding from the low- and high-fiber diets to achieve an increasing gradient with DF.

**Table 1.** Ingredients of the two experimental diets (as fed)

Item	Low fiber	High fiber
Ingredient, g kg^−1^ as fed		
Wheat	820	611
Barley	100	100
Sugar beet pulp	—	210
Soybean meal	27.8	34.1
Molasses	10	10
Leci E Basic^1^	5	5
Palm oil	5	5
Calcium carbonate	13.1	7.35
Sodium chloride	5.29	4.53
Monocalcium phosphate	5.06	5.08
l-Lys	1.93	1.31
l-Thr	0.6	0.52
Titaniumdioxide	3	3
Vitamin and mineral premix^2^	3.222	3.112

^1^Phospolipids, free fatty acid, and triglycerides from rapeseed oil (Evilec Aps, Kolding, Denmark).

^2^Supplemented vitamins and minerals per kilogram of diet: 12,000 IU retinol, 2,000 IU 25-hydroxy vitamin D3 (Hy-D, DSM Nutitional Products, Basel, Switzerland), 100 mg α-tocopherol, 5.00 mg phylloquinone, 2.0 mg thiamin, 0.04 mg cyanocobalamin, 8.00 mg riboflavin, 5.00 mg pyridoxine, 0.60 mg biotin, 25.0 mg d-pantothenic acid, 5.00 mg folic acid, 30.0 mg niacin, 85 mg Choline Extra (choline from a herbal source with no risk of chemical reactions with vitamins [AB Neo A/S, Videbæk, Denmark]), 86.0 mg iron (FeSO_4_), 64.0 mg iron fumerate, 50.0 mg manganese (MnO), 50.0 mg zinc (ZnSO_4_), 50.0 mg zinc chelated (Availa Zinc, Zinpro Corporation, Eden Praire, MN), 15.0 mg cupper as CuSO_4_, 2.00 mg iodine (Ca(IO_3_)_2_), 0.25 mg selenium (Na_2_SeO_3_), 0.15 mg organic selenium (l-selenomethionine, Excential Selenium 4000, Orffa, Breda, The Netherlands), and 1,500 FTU phytase (Ronozyme HiPhos GT, DSM Nutritional Products, Basel, Switzerland).

The animals were fed twice daily (0900 and 1400 hours), and feed leftovers, if any, were collected. Titanium dioxide (**TiO**_**2**_) was added to the diet as an indigestible marker to quantify the digestibility of nutrients. All sows were fed the allocated diet until they were moved to the farrowing unit on day 108.

#### Feeding strategy

Sows were allocated to one of three feeding strategies (**FS**), depending on their BF level (SEGES Innovation, Denmark) at mating; High FS (<13 mm), medium FS (13 to 16 mm), and low FS (> 16 mm) and the FS for individual sows was reconsidered using the same criteria at day 30. From day 60 and onwards, all animals were fed the same FS. The feed was supplied according to Danish feed units for sows, which are closely related to net energy basis (Patience, 2012). Converting to metabolizable energy (**ME**), the high FS sows were fed 55.4 ME/d from days 0 to 30, medium FS sows were supplied 36.9 ME/d, and low FS sows 30.8 ME/d. From days 31 to 60, high FS sows were fed 36.9 ME/d, medium FS sows 32.0 ME/d, and low FS sows 27.1 ME/d. From days 61 to 84, all sows were fed 32.0 ME/d and from days 85 to 108, all sows were fed 43.1 ME/d.

### Experimental procedure

The experiment consisted of two periods with detailed studies, of which days 0 to 30 represent early gestation and days 30 to 60 represent mid-gestation. The subsequent third period, late gestation, focused only on feed intake and changes in BF, BW, and body composition.

#### Sampling

The animals were weighed, and BF scanned at days 0, 30, 60, and 108. Backfat scannings were performed using Lean-Meater (Renco Corp., Minneapolis, MN) at the last rib and 6 cm from the spine, known as P2 BF. A dot was tattooed to ensure repeated measurements were carried out at the same spot at each subsequent sampling. The mean value of six scannings (three on each side) was used to record the BF.

Blood was drawn 4 h after feeding by puncturing the jugular vein at days 0 (only serum), 30, and 60. About 9 mL of blood was collected for harvesting plasma in heparinized vacutainer tubes (Grein Bio-One, GmbH, Kremsmünster, Austria), and the plasma was stored on ice until centrifuging. Moreover, 4 mL of blood was collected in vacutainers without anticoagulant, which was left to clot for a minimum of 6 h before centrifugation and harvest of serum. All samples were centrifuged for 10 min at 1,558 × *g* at 4 °C. Plasma and serum samples were stored at −20 °C for later analysis.

#### Deuterium oxide enrichment

On days 0, 30, 60, and 108, the deuterium oxide (D_2_O) technique was used to assess body pools and of fat and protein according to Rozeboom et al. (1994), which were used to calculate the retention of fat and protein. The D_2_O space in sows was measured as described by Theil et al. (2002b). To determine the D_2_O background level, a urine sample was taken prior to enrichment and stored at −20 °C. Enrichment was done, just before feeding, intra muscularly in the neck or thigh (1S8G, 40-mm needle, 10-mL syringe) with a 40% deuterium oxide (Sigma-Aldrich, MO) and 60% saline (9-mg NaCl/mL; B. Braun Melsungen AG, Melsungen, Germany) solution, by injecting 0.0425 g solution per kilogram live weight. Blood samples were then subsequently collected 4 h after feeding and enrichment, when D_2_O was equilibrated with body water and serum obtained for further analysis. Deuterium oxide is a labeled tracer water isotope and in general, the D_2_O technique is based on the principle that water occupies the fat-free body mass in a relatively fixed fraction.

#### Urine and fecal samples

Feces and urine samples were collected on days 30 and 60. A fresh fecal sample was collected and frozen for further analysis. Urine was collected for 6 h during the daytime using a urinary balloon catheter (Teleflex medical, Kamunting, Malaysia). A stopper in the catheter ensured urine stayed inside the urinary bladder, which was emptied every second hour into a plastic container, which was immediately closed with a lid and kept cold, in a cooling room, until collection was completed. The amount of urine was registered, and a pooled subsample was stored at −20 °C until analysis.

#### Heart rate and feed samples

The heart rate was measured on days 15 and 45 for four consecutive hours, initiated when all sows had completed their morning meal, with a tracking system (Polar Team Pro GPS tracking system, Polar, Ballerup, Denmark) mounted on an elastic band, which were fitted around the belly of the sow just behind the front legs.

Representative feed samples were collected every third week and stored at −20 °C and pooled prior to analysis.

### Chemical analysis

#### Feed and feces

Both feed and fecal samples were analyzed for dry matter (**DM**), ash, total nonstarch polysaccharides (**NSP)**, nitrogen (**N**), gross energy (**GE**), and TiO_2_, and the feed also for Klason lignin. Duplicate analyses were performed on feed samples, whereas single analysis was performed on feces. The analyses of amino acids, N, crude fat, vitamins, and minerals were done according to the Official Journal of the European Union (EU; 152/2009), starch, total, soluble and insoluble NSP, and Klason lignin according to Bach Knudsen (1997), GE using an Automatic Isoperibol Calorimetry system (Parr Instrument Company, Moline, IL, USA), and TiO_2_ as described by Short et al. (1996). Nitrogen was analyzed according to the Dumas method (Hansen, 1989) on a Vario Max CN Element analyzer (Elementar Analysensytem GmbH, Langenselbold, Germany) using aspartic acid as a calibrating standard, and the concentration of crude protein (**CP**) calculated as N × 6.25.

#### Plasma and urine

To determine plasma concentrations of glucose, lactate, triglycerides, and urea, standard assays from Siemens Diagnostics (Siemens Diagnostics Clinical Methods for ADVIA 1650) were applied and quantified using an auto-analyzer (ADVIA 1650 Chemistry System, Siemens Medical Solution, Tarrytown, NY). Nonesterified fatty acid (**NEFA**) was determined using the Wako, NEFA C ACS-ACOD assay method (Wako Chemicals GmbH, Neuss, Germany) and quantified using an auto-analyzer (ADVIA 1650 Chemistry System, Siemens Medical Solution). The content of N in urine was determined by the Kjeldahl method (Method 984.13; AOAC Int., 2000) using a KjelTecTM 2400 (Foss, Hillerød, Denmark). The denoted atomic fraction (**AF**) of the D_2_O space was measured by isotopic ratio mass spectrometry (Delta S; Finnigan MAT, Bremen, Germany), after ultrafiltration and reduction to free hydrogen, as described by Theil et al. (2002b).

### Calculations and statistical analysis

#### Deuterium oxide

From the AF, D_2_O enrichment in infusate and in urine before (AF_P0_) and in serum after enrichment (AF_P1_) the D_2_O space was calculated as


$$\begin{array}{l} D_{2}O\;space[mole]=\frac{Injected\;D_{2}O\;[\;g\;]\;\;}{Molecular\;weight\;of\;injected\;D_{2}O\;[\;g/mol\;]\;\;}\times \frac{AF_{infusate}-AF_{p0}}{AF_{p1}-AF_{p0}}, \end{array}$$

$$\begin{array}{l} D_{2}O\;space\;[kg]=\;\frac{D_{2}O\;space\;\;[\;mole\;]\;\times 18.015\;[g/mol]}{1,000\;[g/kg]}. \end{array}$$

The body pools of protein and fat were then calculated based on formulas for Yorkshire × Landrace gilts as reported by Rozeboom et al. (1994)


$$\begin{array}{l} Body\;protein\;\;[\;kg\;]\;=1.3+0.103\times BW\;\;[\;kg\;]\;+0.092\times D_{2}O\;space\;\;[\;kg\;]\;-0.108\times BF\;\;[\;mm\;], \end{array}$$

$$\begin{array}{l} Body\;fat\;\;[\;kg\;]\;=-7.7+0.649\times BW\;\;[\;kg\;]\;-0.610\times D_{2}O\;space\;[\;kg\;]\;+0.299\times BF\;\;[\;mm\;]\;. \end{array}$$

Retention of body protein (**RP**) and fat (**RF**) was then calculated as


$$\begin{array}{l} Retention\;\;[\;kg/d\;]\;=\frac{\left(Final\;body\;pool\;\;[\;kg\;]\;-initial\;body\;pool\;\;[\;kg\;]\;\right)}{Days}. \end{array}$$

Retained energy (**RE**) was calculated using the data obtained using the D_2_O method, assuming that 1 kg of protein and fat corresponds to 23.9 and 39.8 MJ, respectively (Theil et al., 2020):


$$\begin{array}{l} RE=RP\;\;[\;kg\;]\;\times 23.9\;[MJ/kg]+RF\;\;[\;kg\;]\;\times 39.8\;[MJ/kg]. \end{array}$$

Similarly, retained energy as protein (**RPE**) and retained energy as fat (**RFE**) were calculated using the same energetic constants and expressed relative to realized ME intake.

#### Digestibility

Digestibility of nutrients was measured as apparent total tract digestibility (**ATTD**) using TiO_2_ as a marker, and calculated (with N used as example) as follows:


$$\begin{array}{l} ATTD\;N[\%]=100-\left(100\times \frac{Diet\;TiO_{2}[\%]}{Fecal\;TiO_{2}[\%]}\times \frac{Fecal\;N[\%]}{Diet\;N[\%]}\right). \end{array}$$

An average of TiO_2_ in each batch was used to calculate digestibility for each specific sow and day.

#### Nitrogen balances

The N balance was calculated as N intake minus urinary and fecal N outputs, as follows:


$$\begin{array}{l} Total\;N\;retention\;[g/d]=N\;intake\;[g/d]-(Urinary\;N\;[g/d]+Fecal\;N\;[g/d]). \end{array}$$

#### Energy balances

Retained energy (RE_GE_) was calculated as GE intake minus urine GE, fecal GE, energy lost in methane, and total heat energy (**HE**) as described below:


$$\begin{array}{l} Urine\;GE\;[MJ/d]=\frac{Urine\;N\;[g/d]\times 50.4\;[kJ/g]}{1,000\;[kJ/MJ]}. \end{array}$$

It was assumed that urine contained 50.4 kJ per g of N (Theil et al., 2002a, 2004), and that N lost through urine during 6 h was representative of the daily output.

Gross energy in methane was calculated, according to Jørgensen et al. (2011) using the digestibility and content of NSP in the feed:


$$\begin{array}{l} Methane\;GE\;output[MJ/d]=\frac{0.0628+0.00488\times \left(\;\;\;NSP\;[g/kg\;DM]\times (ATTD\;NSP\;[\%]/100)\right)}{100}\times GE\;intake\;[MJ/d]. \end{array}$$

The HE was calculated according to Krogh et al. (2018), using the mean heart rate recorded:


$$\begin{array}{l} HE\;[MJ/d]=0.323\;\left[\frac{MJ}{d}/bpm\right]\times average\;heartrate\;\;[\;bpm\;]\;-2.4\;[MJ/d]. \end{array}$$

Then, the RE_GE_ was calculated as follows:


$$\begin{array}{l} RE_{GE}\;[MJ/d]=GE_{Intake}\;[MJ/d]-(Fecal\;GE\;output\;\;[\;MJ/d\;]\;+Urine\;GE\;output\;\;[\;MJ/d\;]\;+CH_{4}E\;output\;\;[\;MJ/d\;]\;+HE\;\;[\;MJ/d\;]\;). \end{array}$$

### Statistical analysis

The statistical analysis was performed using a MIXED model in the SAS software (version 9.4, SAS Institute Inc., Cary, NC, 2012). The following model was applied to analyze all data:


$$\begin{array}{l} Y_{ijk}=\mu +\alpha_{i}+\beta_{j}+\tau_{k}+\varepsilon_{ijk}. \end{array}$$

Where *Y*_*ijk*_ is the response variable, μ is the overall mean, α_*i*_ is the fixed effect of treatment (0%, 33%, 67%, and 100% inclusion of the high-fiber diet), β_*j*_ is the fixed effect of feeding strategy (high, medium and low), τ_*k*_ is the random effect of block (1 and 2) and ε_*ijk*_ is the residual error component, which was assumed to be normally distributed N (0, σ^2^). Within the model, analysis of variance (**ANOVA**), linear effect for dietary treatments was tested, whereas FS was only tested using the ANOVA. Mean values are presented as least square means ± SEM, where the highest SEM for treatment and FS, respectively, are shown. Effects were considered significant at *P* ≤ 0.05 and as tendencies when 0.05 < *P* ≤ 0.10. Interaction between DF and FS were tested, but none were found significant and hence not reported.

## Results

### Dietary treatments

The DF increased from 119 g/kg in the low-fiber diet to 217 g/kg in the high-fiber diet, and concomitantly the calculated dietary content of ME decreased from 13.5 MJ/kg in the low-fiber to 12.7 MJ/kg in the high-fiber diet. The CP content increased 4% from 115 g/kg in the low DF diet to 120 g/kg in the high DF diet, while lysine decreased by 7% from 5.47 g/kg to 5.09 g/kg (Table 2).

**Table 2.** Analyzed chemical composition (as fed) of experimental diets

Item^1^	0	33	67	100
Chemical composition, g × kg^−1^				
DM	879	878	877	877
Ash	37.8	38.8	39.9	41.0
Fat	28.5	28.8	29.2	29.5
Starch	541	491	440	391
Soluble (S-NSP)	17.8	33.9	50.6	66.7
Insoluble nonstarch polysaccharides (I-NSP)	85.8	97.3	109.1	120.5
Klason lignin	15.9	20.5	25.3	30.0
Dietary fiber	119	152	185	217
EDOM^2^	905	901	897	893
GE, MJ/kg	15.6	15.6	15.6	15.5
Energy, MJ ME/kg^3^	13.5	13.2	13.0	12.7
Energy, MJ net energy/kg^4^	10.3	10.0	9.8	9.6
Energy, Danish Feed Units/kg^5^	1.13	1.10	1.07	1.04
Titanium dioxide	1.79	1.90	2.01	2.11
Protein and amino acids, g × kg^−1^^6^				
CP	115 (86.0)	117 (87.7)	118 (89.4)	120 (91.1)
Lysine	5.47 (4.2)	5.34 (4.1)	5.21 (4.0)	5.09 (3.9)
Methionine	1.75 (1.3)	1.72 (1.3)	1.69 (1.3)	1.67 (1.3)
Threonine	4.16 (3.1)	4.25 (3.1)	4.33 (3.1)	4.42 (3.1)
Leucine	7.85 (6.1)	7.75 (6.1)	7.64 (6.1)	7.54 (6.1)
Valine	5.08 (3.8)	5.15 (3.8)	5.22 (3.9)	5.29 (3.9)
Minerals, g × kg^−1^				
Sodium	1.85	1.88	1.90	1.93
Potassium	4.91	4.96	5.00	5.05
Magnesium	1.10	1.18	1.27	1.36
Calcium	7.04	6.94	6.85	6.76
Phosphorus	4.15	4.05	3.95	3.86
Trace minerals, mg × kg^−1^				
Iron	281.3	296.2	311.6	326.5
Cupper	22.0	20.9	19.8	18.7
Manganese	66.8	69.5	72.2	74.9
Zinc	121.5	121.7	121.8	122.0

^1^Proportions of a high-fiber diet, and rest refer to low-fiber diet.

^2^Enzyme digestible organic matter.

^3^Calculated from Danish feed units (Theil et al., 2020).

^4^Calculated according to EvaPig (2008).

^5^Danish feeding units for sows: the Danish feed units have potential physiological energy, and this is closely related to net energy (Patience, 2012).

^6^Calculated standardized ileal digestibility values in brackets.

### Feeding strategy

On day 30, 12 sows were moved from the high to the medium FS, 6 sows from the medium to the low FS, 2 sows from the high to the low FS, 1 sow from the low to the medium FS, and the remaining sows stayed at the same FS as in early gestation. As a consequence, only two sows were fed the high feeding strategy in mid-gestation, and due to the low number in that treatment group, they were omitted in the statistical analysis for mid-gestation (days 30 to 60).

### Digestibility

With increasing fiber levels, the digestibility of DM, N, GE, and organic matter decreased linearly in early gestation (*P* < 0.001; Table 3). The same pattern was observed in mid-gestation (*P* < 0.05) although no evidence of a dietary effect on DM digestibility was observed. Digestibility of NSP increased linearly with increasing fiber inclusion in both gestation periods (*P* < 0.001). The FS did not affect the digestibility of nutrients, except the digestibility of NSP, which tended (*P* = 0.06) to be higher in sows fed the medium FS as compared with sows fed the low FS in mid-gestation.

**Table 3.** Apparent total tract digestibility of nutrients and energy in sows fed increasing levels of dietary fiber originating from sugar beet pulp with different feeding strategies in early (days 0 to 30) and mid (days 30 to 60) gestation^1^

Item	Dietary fiber (DF), g × kg^−1^^2^					Feeding strategy (FS)^3^				*P*-value		
	119	152	185	217	SEM							
	0	33	67	100		High	Medium	Low	SEM	DF	FS	Linear
Early gestation												
*n*	13	12	11	10		16	12	18				
Dry matter (DM) digestibility, %	86.5^a^	85.9^a^	85.4^bc^	85.0^c^	0.28	85.6	85.9	85.6	0.27	<0.01	0.68	<0.001
Nitrogen (N) digestibility, %	82.2^a^	80.7^a^	79.2^bc^	78.0^c^	0.54	79.8	80.0	80.3	0.52	<0.001	0.77	<0.001
Nonstarch polysaccharide (NSP) digestibility, %	64.3^d^	70.2^c^	75.6^b^	79.5^a^	0.85	71.1	73.5	72.7	0.82	<0.001	0.13	<0.001
Gross energy (GE) digestibility, %	86.7^a^	85.9^a^	85.1^bc^	84.3^c^	0.31	85.2	85.7	85.5	0.30	<0.001	0.59	<0.001
Organic matter digestibility, %	89.5^a^	88.7^b^	88.2^bc^	87.7^c^	0.22	88.4	88.7	88.6	0.22	<0.001	0.65	<0.001
Mid-gestation												
*n* ^4^	13	10	11	9			19	22				
DM digestibility, %	86.2	86.5	85.7	85.7	0.50		86.3	85.7	0.35	0.54	0.20	0.24
N digestibility, %	82.2^a^	81.4^ab^	79.5^ab^	79.0^b^	0.88		80.9	80.2	0.61	<0.01	0.43	<0.001
NSP digestibility, %	63.3^d^	71.1^c^	75.6 ^b^	80.8^a^	0.87		73.5	71.9	0.60	<0.001	0.06	<0.001
GE digestibility, %	86.4	86.4	85.4	85.2	0.49		86.1	85.5	0.34	0.11	0.24	<0.05
Organic matter digestibility, %	89.2	89.2	88.5	88.4	0.39		89.0	88.6	0.27	0.20	0.27	0.05

^1^Data are least square mean values with their SEM.

^2^Proportions of a high-fiber diet, and rest refer to low-fiber diet.

^3^Feeding strategy at days 0 and 30: backfat level: < 12 mm: high, 12 to 16 mm: medium and > 16 mm: low.

^4^Two sows were omitted (as compared with day 30) due to insufficient number of animals on the High FS.

^a-d^Means within a row with different superscripts differ (*P* ≤ 0.05).

### Sow performance and feed utilization

In early gestation, BF (due to the experimental design) and  BW, body protein, and body fat differed between FS and were lowest for sows on the high FS, intermediate on the medium FS, and highest on the low FS (Table 4). The average daily feed intake (**ADFI**) in early gestation increased linearly from 2.83 kg/d in the low-fiber diet to 3.07 kg/d in the high-fiber diet, and concomitantly the fiber intake increased from 346 g/d to 662 g/d (*P* < 0.001). The fiber intake also increased in mid-gestation (*P* < 0.001). In both early- and mid-gestation, ADFI, energy intake, and fiber intake were highest in sows fed the high FS, intermediate in the medium FS, and lowest in the low FS group (*P* < 0.001).

**Table 4.** Initial parameters, intake (as fed), and performance in early (days 0 to 30), mid (days 30 to 60), and late (days 60 to 108) gestation in sows fed increasing levels of dietary fiber originating from sugar beet pulp with different feeding strategies^1^

Item	Dietary fiber (DF), g × kg^−1^^2^					Feeding strategy (FS)^3^				*P*-value		
	119	152	185	217	SEM							
	0	33	67	100		High	Medium	Low	SEM	DF	FS	Linear
*Initial parameters and sow performance*												
*n*	13	12	11	10		16	12	18				
Parity	2.5	2.5	2.5	2.5	0	2.5	2.5	2.5	0			
Sow BW day 0, kg	231	224	234	227	8.22	214^c^	224^b^	248^a^	7.90	0.83	<0.01	0.98
Sow BF day 0, mm	14.0	13.9	14.7	14.5	0.58	11.1^c^	14.1^b^	17.6^a^	0.56	0.72	<0.001	0.37
Body protein day 0^4^, kg	37.2	35.3	36.7	35.3	1.36	34.9	35.0	38.5	1.23	0.54	<0.05	0.43
Body fat day 0^4^, kg	57.1	58.9	62.0	57.2	4.16	49.7^b^	56.4^b^	70.3^a^	3.76	0.73	<0.001	0.78
Total born piglets, n	19.8	18.7	21.7	23.1	1.60					0.20		0.06
Birth weight total litter, kg	24.3	24.8	25.6	26.1	1.60					0.82		0.35
*Early gestation*												
ADFI, kg/d	2.83^b^	2.88^b^	2.96^ab^	3.07^a^	0.05	4.01^a^	2.66^b^	2.14^c^	0.05	<0.01	<0.001	<0.001
Fiber intake, g/d	346^d^	444^c^	557^b^	662^a^	13.8	674^a^	464^b^	368^c^	13.2	<0.001	<0.001	<0.001
BW gain, kg/d	0.417^b^	0.478^b^	0.705^a^	0.596^a^	0.065	0.957^a^	0.491^b^	0.199^c^	0.062	<0.01	<0.001	<0.01
BF gain, mm/d	0.046	0.055	0.054	0.052	0.010	0.102^a^	0.045^b^	0.008^c^	0.009	0.90	<0.001	0.68
RP^4^, kg/d	0.061^b^	0.072^ab^	0.110^a^	0.088^ab^	0.014	0.129^a^	0.081^b^	0.038^c^	0.013	<0.05	< 0.0001	<0.05
RF^4^, kg/d	0.125	0.145	0.187	0.199	0.054	0.374^a^	0.110^b^	0.008^b^	0.049	0.63	<0.001	0.21
BW gain:feed ratio, kg/kg_feed_	0.139^b^	0.146^b^	0.238^a^	0.190^ab^	0.025	0.266^a^	0.179^b^	0.089^c^	0.024	<0.05	<0.001	<0.05
BF gain:feed ratio, mm/kg_feed_	0.014	0.016	0.017	0.016	0.004	0.028^a^	0.016^b^	0.003^c^	0.004	0.96	<0.001	0.66
RP:feed ratio^4^, kg/kg_feed_	0.021^b^	0.023^ab^	0.038^a^	0.029^ab^	0.006	0.036^a^	0.030^ab^	0.017^b^	0.005	<0.05	<0.01	0.06
RF:feed ratio^4^, kg/kg_feed_	0.038	0.045	0.057	0.061	0.021	0.104^a^	0.041^b^	0.006^b^	0.019	0.80	<0.001	0.34
RPE^5^, % of ME_Intake_	3.7^b^	4.7^ab^	7.2^a^	4.8^ab^	1.1	6.4	5.5	3.4	1.0	<0.05	<0.05	0.14
RFE^5^, % of ME_Intake_	10	15	17	20	7.7	31^a^	13^b^	2^b^	7.2	0.75	<0.01	0.30
RE^5^, % of ME_Intake_	14	20	25	25	7.5	38^a^	18^b^	6^b^	7.1	0.56	<0.01	0.21
*Mid-gestation*												
*n* ^6^	12	10	11	8			19	22				
ADFI, kg/d	2.12	2.13	2.15	2.18	0.05		2.36^a^	1.93^b^	0.032	0.78	<0.001	0.31
Fiber intake, g/d	253^d^	329^c^	407^b^	476^a^	8.3		402^a^	331^b^	17.9	<0.001	<0.001	<0.001
BW gain, kg/d	0.37	0.49	0.36	0.45	0.082		0.49	0.34	0.057	0.53	0.08	0.81
BF gain, mm/d	0.016	0.023	0.003	0.023	0.012		0.032	0.000	0.007	0.35	<0.01	0.96
RP^4^, kg/d	0.042	0.060	0.055	0.060	0.017		0.068	0.041	0.012	0.79	0.11	0.45
RF^4^, kg/d	0.23	0.28	0.20	0.19	0.058		0.30	0.14	0.039	0.58	<0.01	0.40
BW gain:feed ratio, kg/kg_feed_	0.18	0.23	0.16	0.21	0.038		0.21	0.18	0.026	0.50	0.36	0.85
BF gain:feed ratio, mm/kg_feed_	0.020	0.027	0.025	0.029	0.008		0.029	0.022	0.005	0.84	0.36	0.45
RP:feed ratio^4^, kg/kg_feed_	0.007	0.009	0.000	0.011	0.005		0.014	0.000	0.003	0.35	<0.01	1.00
RF:feed ratio^4^, kg/kg_feed_	0.108	0.137	0.090	0.091	0.029		0.138	0.075	0.019	0.50	<0.05	0.40
RPE^5^, % of ME_Intake_ Retention_protein_/ME_Intake_	3.7	5.2	4.7	5.4	1.5		5.4	4.1	1.0	0.79	0.37	0.45
RFE^5^, % of ME_Intake_	33	42	27	28	9.0		42^a^	23^b^	6.0	0.56	<0.05	0.42
RE^5^, % of ME_Intake_	37	47	32	33	9.0		48^a^	27^b^	6.0	0.55	<0.05	0.49

^1^Data are least square mean values with their SEM.

^2^Proportion of a high-fiber diet, and rest refer to low-fiber diet.

^3^Feeding strategy at days 0 and 30: backfat level: < 12 mm: high, 12 to 16 mm: medium and > 16 mm: low.

^4^Retained protein and fat, calculated from the deuterium oxide (D_2_O) dilution technique.

^5^Retained energy as protein: retained protein × 23.9 MJ/kg and retained energy as fat: retained fat × 39.8 MJ/kg as percentage of experimentally found metabolizable energy (ME) intake (Table 6).

^6^Two sows were omitted (as compared with days 0 to 30) due to insufficient number of animals on the High FS.

^a-d^Means within a row with different superscripts differ (*P* ≤ 0.05).

**Figure 1.**
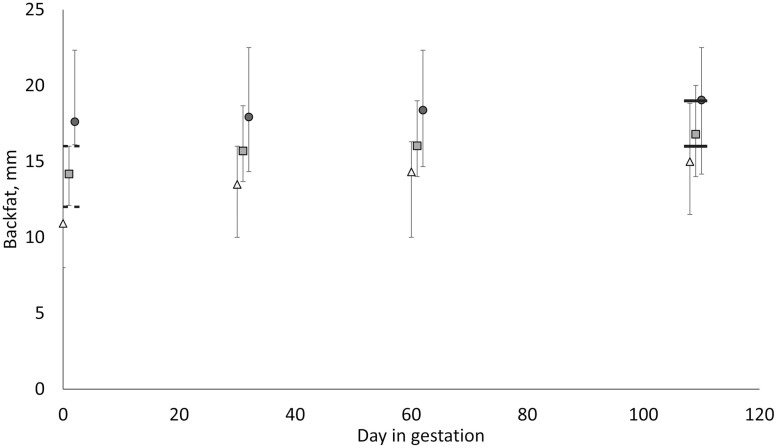
Backfat (BF) thickness on days 0, 30, 60, and 108 after service, the feeding strategy groups, low (●), medium (■), and high (▲) are the groups defined at day 0, the limits defining the normal feeding strategy group at day 0 (12 to 16 mm BF) are represented by two dashed lines, whereas the target zone for all sows at day 108 (16 to 19 mm BF) is represented by two solid lines.

In early gestation, BW gain (*P* < 0.01) and RP (*P* < 0.05) increased linearly with increased fiber inclusion, and RF increased 59% (from 0.125 to 0.199 kg/d) although that change was not statistically significant. In mid and late gestation, no differences in BW gain, BF gain, RP, and RF were observed across the dietary treatments. In early gestation, sows fed the high FS had the greatest increase in BF, sows fed the medium FS had a lower increase in BF and sows fed the low FS hardly gained BF in this period (*P* < 0.001). From mating until sows entered the farrowing unit (day 108), sows gained 4.1, 2.6, and 1.4 mm in BF, respectively (Figure 1), when considering the feeding strategies applied from days 0 to 30. Body weight gain-to-feed ratio and RP-to-feed ratio were lowest in the low fiber treatment and increased linearly with increasing DF (*P* < 0.05) in early gestation. Retained protein energy as percentage of ME_Intake_ was greatest, with 7.2%, when including 185 g DF/kg compared to 3.7% when no SBP was included (*P* < 0.05). In early gestation, the high FS had the highest BW, BF, and RF-to-feed ratio (*P* < 0.001) and RP-to-feed ratio (*P* < 0.01).

### Metabolites

In both early- and mid-gestation, plasma urea decreased linearly when fiber intake increased (*P* < 0.05; Table 5). The plasma NEFA was highest (*P* < 0.01) in both early- and mid-gestation in sows fed the low FS, and plasma urea was highest in sows fed the high FS in early gestation (*P* < 0.001), while no difference was observed in mid-gestation for plasma urea.

**Table 5.** Plasma metabolites in sows fed increasing levels of dietary fiber originating from sugar beet pulp with different feeding strategies in early (day 30) and mid (day 60) gestation^1^

Item	Dietary fiber (DF), g × kg^−1^^2^					Feeding strategy (FS)^3^				*P*-value		
	119	152	185	217	SEM							
	0	33	67	100		High	Medium	Low	SEM	DF	FS	Linear
*Gestation day 30*												
*n*	13	12	11	10		16	12	18				
NEFA, µekv./L	44.6	45.4	66.7	53.5	8.5	36.0^b^	52.8^ab^	68.8^a^	7.8	0.16	<0.01	0.14
Glucose, mmol/L	4.22	4.42	4.57	4.24	0.14	4.52	4.25	4.31	0.13	0.20	0.33	0.61
Urea, mmol/L	3.17	3.00	2.81	2.79	0.13	3.52^a^	2.85^b^	2.46^c^	0.13	0.11	<0.001	<0.05
Lactate, mmol/L	2.05^b^	3.28^a^	1.48^b^	1.54^b^	0.40	2.17	2.13	1.96	0.38	<0.01	0.88	0.05
Triglyceride, mmol/L	0.34^b^	0.44^a^	0.42^a^	0.35^b^	0.02	0.41	0.39	0.36	0.02	<0.01	0.21	0.72
*Gestation day 60*												
*n* ^4^	13	10	11	9			19	22				
NEFA, µekv./L	58.0	58.7	52.2	60.0	10.6		44.8^b^	79.4^a^	7.05	0.77	<0.01	0.34
Glucose, mmol/L	4.54	4.48	4.62	4.55	0.12		4.42	4.57	0.08	0.94	0.20	0.64
Urea, mmol/L	2.68^a^	2.44^ab^	2.40^ab^	2.19^b^	0.11		2.49	2.37	0.08	<0.05	0.28	<0.01
Lactate, mmol/L	1.89	1.58	1.22	1.71	0.26		1.64	1.56	0.19	0.20	0.74	0.33
Triglyceride, mmol/L	0.39	0.42	0.42	0.39	0.03		0.40	0.41	0.02	0.75	0.66	0.87

^1^Data are least square mean values with their SEM.

^2^Proportions of a high-fiber diet, and rest refer to low-fiber diet.

^3^Feeding strategy at days 0 and 30: backfat level: < 12 mm: high, 12 to 16 mm: medium and > 16 mm: low.

^4^Two sows were omitted (as compared with day 30) due to insufficient number of animals on the High FS.

^a-c^Means within a row with different superscripts differ (*P* ≤ 0.05).

### Balances of N and GE

**Figure 2.**
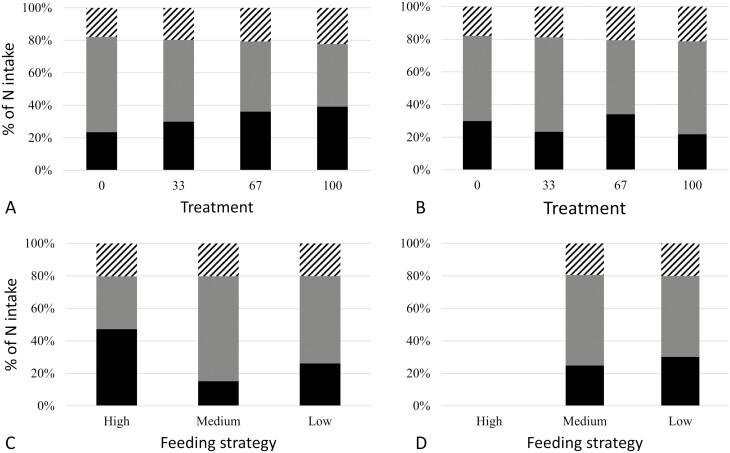
Fecal N output (striped), urine N output (gray), and N retention (black) as percentage of intake, in early (A) and mid (B) gestation, depending on treatment, with proportion of high-fiber diet (the rest is low-fiber diet) and depending on feeding strategy in early (C) and mid (D) gestation.

N intake, N loss in feces (*P* < 0.001; Table 6), and total N retention (*P* < 0.05) increased linearly with increasing fiber intake in early gestation. Expressed relative to N intake, fecal N output only accounted for 18% to 22% in the low- and high-fiber diets, respectively, N lost through urine decreased from 59% to 38%, and N retention increased from 23% to 39% with increasing inclusion level of fiber (Figure 2A). In mid-gestation, N intake (*P* < 0.05) and fecal N output (*P* < 0.001) increased linearly with increasing fiber intake, whereas total N retention and urinary N loss did not differ across treatments.

**Table 6.** Realized nitrogen (N) and gross energy (GE) intake, output, and total retention (RE) in sows fed increasing levels of dietary fiber originating from sugar beet pulp with different feeding strategies in early (days 0 to 30) and mid (days 30 to 60) gestation^1^

Item	Dietary fiber (DF), g × kg^−1^^2^					Feeding strategy (FS)^3^				*P*-value		
	119	152	185	217	SEM							
	0	33	67	100		High	Medium	Low	SEM	DF	FS	Linear
*Early gestation*												
*n*	13	12	11	10		16	12	18				
Realized N intake, g/d	52.2^c^	53.9^bc^	56.2^b^	58.9^a^	0.9	75.4^a^	50.3^b^	40.3^c^	0.9	<0.001	<0.001	<0.001
Urine N output, g/d	30.6	26.9	24.3	22.6	4.3	24.6	32.4	21.3	4.2	0.51	0.12	0.14
Fecal N output, g/d	9.3^c^	10.5^bc^	11.6^b^	13.1^a^	0.45	15.3	10.1	8.0^c^	0.31	<0.001	<0.001	<0.001
Total N retention^4^, g/d	12.2	16.0	20.3	23.0	3.9	35.7^a^	7.6^b^	10.3^b^	3.8	0.18	<0.001	<0.05
Heart rate, average, bpm.	96.8	87.3	93.5	90.8	5.3	95.4	91.4	89.6	5.1	0.57	0.66	0.58
GE intake, MJ/d	44.2^b^	44.9^b^	46.1^ab^	47.8^a^	0.76	62.5^a^	41.5^b^	33.3^c^	0.78	<0.01	<0.001	<0.001
Urine GE output^5^, MJ/d	1.5	1.4	1.2	1.1	0.22	1.2	1.6	1.1	0.21	0.51	0.12	0.14
Fecal GE output, MJ/d	5.9^c^	6.4^b^	6.9^a^	7.5^a^	0.18	9.2^a^	6.0^b^	4.8^c^	0.17	<0.001	<0.001	<0.001
Methane GE output^6^, MJ/d	0.2^d^	0.3^c^	0.4^b^	0.5^a^	0.01	0.5^a^	0.3^b^	0.3^c^	0.0	<0.001	<0.001	<0.001
ME^7^, MJ/d	36.5^b^	36.6^b^	37.7^a^	38.5^a^	0.57	51.8^a^	33.5^b^	26.6^c^	0.64	<0.05	<0.001	<0.01
HE total^8^, MJ ME/d	28.9	25.8	27.8	26.9	1.7	28.4	27.1	26.5	1.6	0.57	0.66	0.58
RE_GE_^9^, MJ/d	7.9	8.8	9.7	11.5	2.2	23.9^a^	5.4^b^	−0.8^c^	1.99	0.61	<0.001	0.19
Realized dietary ME^10^, MJ/kg	12.9	12.8	12.7	12.6	0.08	12.9^a^	12.6^b^	12.7^ab^	0.08	0.13	<0.05	<0.05
*Mid-gestation*												
*n* ^11^	12	10	11	8			19	22				
Realized N intake, g/d	39.0	39.7	40.8	41.8	0.87		44.4^a^	36.3^b^	0.60	0.08	<0.001	<0.05
Urine N output, g/d	20.4	23.0	18.5	23.8	3.1		24.7^a^	18.1^b^	2.2	0.53	<0.05	0.69
Fecal N output, g/d	7.0^b^	7.5^bc^	8.4^ab^	8.9^a^	0.45		8.6^a^	7.3^b^	0.31	<0.01	<0.01	<0.001
Total N retention^4^, g/d	11.7	9.3	13.9	9.1	2.8		11.0	11.0	1.9	0.50	0.98	0.84
Heart rate, average, bpm.	100.0	92.5	90.9	85.3	5.7		96.8	87.5	11.9	0.26	0.11	0.06
GE intake, MJ/d	33.1	33.1	33.5	33.9	0.72		36.7^a^	30.1^b^	0.50	0.85	<0.001	0.39
Urine GE output^5^, MJ/d	1.0	1.2	0.9	1.2	0.16		1.2^a^	0.9^b^	0.11	0.53	<0.05	0.69
Fecal GE output, MJ/d	4.5	4.6	4.9	5.1	0.21		5.2^a^	4.4^b^	0.14	0.13	<0.001	<0.05
Methane GE output^6^, MJ/d	0.2^d^	0.2^c^	0.3^b^	0.4^a^	0.0		0.3^a^	0.2^b^	0.0	<0.001	<0.001	<0.001
ME^7^, MJ/d	27.4	27.2	27.4	27.3	0.56		30.1^a^	24.6^b^	0.39	0.99	<0.001	0.91
HE total^8^, MJ ME/d	29.9	27.5	27.0	25.2	1.8		28.9	25.9	1.3	0.25	0.11	0.06
RE_GE_^9^, MJ/d	−2.5	−0.4	0.4	2.1	1.91		4.5	1.5	1.4	0.31	0.19	0.07
Realized dietary ME^10^, MJ/kg	12.9	12.9	12.7	12.6	0.11		14.2	14.3	0.14	0.09	0.81	<0.05

^1^Data are least square mean values with their SEM.

^2^Proportion of a high-fiber diet, and rest refer to low-fiber diet.

^3^Feeding strategy at days 0 and 30: backfat level: <12 mm: high, 12 to 16 mm: medium, and >16 mm: low.

^4^N intake − (Urine N output + Fecal N Output).

^5^Assuming that energy in urine from sows contains 50.4 kJ/g N (Theil et al., 2002a, 2004).

^6^0.0628 + 0.00488 × fermented fiber, g/kg dry matter (Jørgensen et al., 2011).

^7^GE intake − (Urine GE output + Methane GE output + Feces GE output).

^8^Calculated using the heart rate and heat energy (HE) relationship (Krogh et al., 2018).

^9^ME intake—HE total.

^10^(GE intake − [Urine GE output + Methane GE output + Fecal GE output])/average daily feed intake.

^11^Two sows were omitted (as compared with day 30) due to insufficient number of animals on the High FS.

^a-d^Means within a row with different superscripts differ (*P* ≤ 0.05).

Heart rate tended to decrease in mid-gestation in response to increased fiber levels (*P* = 0.06), and the same pattern was observed in early gestation, although there was no statistical evidence of a dietary response.

In early gestation, the GE intake, fecal GE output, and methane GE output increased linearly (*P* < 0.001) with increasing fiber levels. The total ME increased with increasing fiber inclusion from 36.5 MJ/d in the low-fiber diet to 38.5 MJ/d in high-fiber diet (*P* < 0.001). In mid-gestation, fecal and methane GE output increased linearly (*P* < 0.05), while HE tended to decrease linearly with increasing fiber inclusion (*P* = 0.06) giving rise to a tendency of increased total energy retention from −2.5 MJ/d in the low-fiber diet to 2.1 MJ/d in the high-fiber diet (*P* = 0.07). Regarding FS in early gestation, GE intake, fecal GE output, methane GE output, ME, and total energy retention (*P* < 0.001) were highest in the high FS, intermediate in the medium, and lowest in the low FS group. In mid-gestation, GE intake, Fecal GE output, methane GE output, ME (*P* < 0.001), and urine GE output (*P* < 0.05) were higher in medium FS and lower in low FS while retained energy did not differ between FS. The realized dietary ME did not differ across dietary treatments and amounted to 12.6 to 12.9 MJ ME/kg feed in both early- and mid-gestation.

## Discussion

### Restoring body condition

High feed intake restored BF in sows with inadequate BF almost to the targeted level (16 to 19 mm BF), while a low feed intake maintained the BF level fairly constant in sows that had adequate BF at mating. Thus, restoring BF by adjusting the feeding level was successful, as the sows fed the high feeding level rapidly increased their BF in early gestation (+ 2.6 mm from days 0 to 30 and + 4.1 mm during the entire gestation), while fat sows fed the low feeding strategy gained least BF (+ 0.3 mm from days 0 to 30 and + 1.4 mm during the entire gestation). This is in line with Young et al. (2004) who found that feeding according to BF thickness resulted in more sows ending up within their target zone (17 to 21 mm BF in their study) at farrowing as compared with visual scoring of body condition. This is also supported by Maes et al. (2004), who found that BF measurements and visual scoring were only moderately correlated. In Denmark, it is common to differentiate the FS depending on BF level at mating and supply feed within the range of 2.2 to 4.3 kg/d from days 0 to 30 with the overall aim of reaching 16 to 19 mm of BF when sows enter the farrowing unit around day 108 (Pedersen, 2020). On day 30, the recommended feed supply was lowered for all FS as compared with early gestation to attenuate retention of BF. From days 60 until 84 of gestation, it is recommended to supply all sows with 2.2 kg/d, and from day 85 and up until sows enter the farrowing unit, the feed supply should be kept constant at 3.3 kg/d (SEGES, 2019). In the current study, reduced variation in BF was observed with progress of gestation but unfortunately the high FS group barely reached the range of 16 to 19 mm BF. Increasing the level of DF from 119 to 217 g/kg by increasing the inclusion level of SBP increased the BW gain linearly from 417 to 596 g/d, and the body fat retention of sows by 59% (from 125 to 199 g/d) based on the D_2_O dilution technique, although the latter did not differ significantly. Increasing the DF concentration at the expense of decreased starch increased the uptake to the portal vein of ketogenic energy metabolites, acetate, and butyrate at the expense of glucose (Serena et al., 2009). These ketogenic metabolites are precursors that can directly be utilized in de novo fat synthesis in anabolic sows (Theil et al., 2020). Therefore, DF most likely favor body fat accretion. In contrast to our expectation, the body protein retention and sow BW gain increased linearly with increasing DF concentration in early gestation. In the lactation period prior to this study, the sows lost on average 8% of their BW, 2% of their body protein pool, and 16% of their body fat pool (Bruun et. al., 2023). Hence the observed protein retention seemed not to be driven by a pronounced need for restoring lost body protein. Two explanations seem to be plausible for the fiber effect on protein retention. One option is that sows fed increasing amounts of fiber ingested more ME, which normally is the limiting factor for protein retention in gestating sows (Dourmad et al., 2008). Another option is that increasing the proportion of energy originating from fiber at the expense of starch attenuates the diurnal fluctuations in energy uptake from the gastrointestinal tract (Serena et al., 2009) thereby improving the utilization of dietary amino acids and CP and, unintendedly, increase lean growth instead of fat retention. Both mechanisms may well have contributed to altering the feed utilization in the sows in the present study and blurred the dietary responses on restored body fat and BF across the four dietary treatments.

### Fiber and HE

Heat energy is influenced by many factors, including live weight, feed intake, feed composition, ambient temperature, stage of gestation, and physical activity (Brouns et al., 1994; Theil et al., 2020). It is indeed a challenge to quantify the HE of gestating sows because physical activity is confined when studied in respiration chambers (Jakobsen et al., 2005). In the current study, the mean heart rate recorded during a 4-h period after completion of the morning meal was used to estimate the daily HE. According to the recorded mean heart rate, the study suggested that the HE could be 7% (NS) and 16% (*P* = 0.06) lower in early- and mid-gestation, respectively, when comparing the low- and high-fiber diets. In agreement with this, Rijnen et al. (2003), reported that group-housed sows had decreased HE when they were fed high levels of SBP. On the other hand, Olesen et al. (2001) reported a numerically greater HE in sows fed a high-fiber mix diet (with fibers originating from SBP, oats, grass pellets, and wheat bran). The higher HE reported for the high-fiber diet reported by Olesen et al. (2001) is in agreement with the theoretical held framework that the handling of a more bulky digesta will require more energy. However, in the case of SBP this effect may be overshadowed by a higher energy efficiency of absorbed SCFA (acetate, propionate, and butyrate) originating from fermentation of fibers in the hindgut compared to glucose from starch. The SCFA give raise to less heat when utilized for de novo fat synthesis as compared with glucose, because of more steps in the metabolic pathway for glucose than for ketogenic SCFA. Thus, 2 of 6 carbons (33%) and 14 of 38 adenosine triphosphate (37%) are lost and correspondingly additional heat is generated when glucose is converted into two molecules of acetyl Coenzyme A (Theil et al., 2020). In contrast, no carbons are lost (and less heat is produced) if acetate or butyrate are precursors for de novo fat synthesis. These aspects are not taken into account in the Danish energy evaluation system because the system only considers the potential energy value of digested nutrients under the assumption that they are completely oxidized (Tybirk et al., 2006). Another reason why HE decreased with increasing fiber inclusion may be due to fermentation heat from the hindgut, which supplies extra intrinsic heat to the sow and possibly reduces the need for HE due to thermoregulation. This is, however, mostly relevant for sows fed around (or below) maintenance, whereas sows fed well above maintenance (e.g., to restore BF) produce so much additional heat due to the anabolic processes that no nutrients need to be oxidized for thermoregulatory purposes. In line with the latter explanation, Ramonet et al. (2000) explored the partitioning of HE between a combined fiber source and a control diet in gestating sows fed around maintenance. In the study, they reported that the thermic effect of feeding (i.e., increased post-prandial HE due to digestion processes) increased when feeding a fiber-rich diet (Ramonet et al., 2000). To fully understand and differentiate the heat production between maintenance, anabolic and fermentation processes, further studies are needed, especially with focus on fat retention.

### Fiber and energy balance in early- and mid-gestation

Total energy retention tended to increase linearly with the inclusion of SBP in the diet in mid-gestation (from days 30 to 60) and the same pattern was observed in early gestation, although no statistical difference was observed. This effect was primarily due to increased intake of ME combined with decreasing energy lost as heat. This is in line with Rijnen et al. (2001), who found a constant total energy retention, in spite of a decrease in ME intake. All groups of sows in the present study metabolized the energy that was expected based on the dietary formulation. The sows metabolized 12.9 and 12.6 MJ ME/kg in the low- and high-fiber diet in early gestation, respectively, which was close to what was expected based on the Danish feed evaluation system (13.5 and 12.7 MJ/kg, respectively).

Plasma NEFA is a good indicator of the energy balance of the animal (Ren et al., 2017), as also reflected by the decreased NEFA with increasing feed intake across feeding strategies. In the current study, the plasma NEFA concentration was not affected by the inclusion level of SBP, which supported the lack of difference of different fiber levels on energy retention.

A benefit of achieving a correct energy value of the sow diets is that the protein (i.e., amino acids) to energy ratio is as close as possible to the National nutrient recommendations (Tybirk et al., 2020). Underestimating the energy value in sow diets can lead to increased protein and fat retention and hence unintended weight gain, which in turn increases the energy requirement for maintenance throughout the remaining life of the sow. Moreover, it may lead to increased risk of overloading the joints with potential consequences on reproductive performance (Prunier et al., 2010) and reduced longevity (Jorgensen and Sorensen, 1998). Increased energy intake, even when provided as energy as in the current study, can increase the protein retention.

### Fiber and impact on feed efficiency and productivity

Sugar beet pulp, compared to other fiber sources, is highly fermentable and thus a well-suited fiber source for gestating sows, as it contains high levels of pectin (uronic acids) and low levels of lignin (Bach Knudsen, 1997). Moreover, SBP is characterized by a high-water binding capacity (Zhou et al., 2018), which increases the gut fill. A high-water binding capacity increases the surface area of the feed matrix in the gastrointestinal tract, allowing easier access for microbial enzymes to reach their substrates in the hindgut (Renteria-Flores et al., 2008; Priester et al., 2020). In the current study, the digestibility of GE and all nutrients except NSP decreased with an increased amount of SBP. This is a consequence of the high ratio of soluble compared to insoluble NSP (Burkhalter et al., 2001) and in agreement with studies of Olesen et al. (2001) and Renteria-Flores et al. (2008) that also showed higher fermentability of fibers in SBP compared with other fiber sources like wheat bran and wheat straw (Renteria-Flores et al., 2008).

In spite of decreasing digestibility of both energy and N, the feed efficiencies were higher when more fibers were included in the diet. That the protein gain per kilogram feed (from days 0 to 30) increased from 119 to 185 g DF/kg emphasizes that the sow’s ability to utilize both N and energy was highly efficient even at high inclusion levels of SBP.

Both a higher energy intake and indication of a more stable diurnal uptake of energy, especially in early gestation, may well affect farrowing parameters as total born because the energy balance influences the embryonic mortality (Zhou et al., 2019; Peltoniemi et al., 2021). In the current study, the inclusion of SBP tended to increase the number of total born piglets, which corroborated the findings of van der Peet-Schwering et al. (2003) who found an increase of 0.5 total born piglets when sows were fed a high-fiber diet throughout three parities. On the other hand, Danielsen and Vestergaard (2001) did not find any impact of feeding a low-fiber control diet or a high-fiber diet based on SBP on number of total born piglets, but they reported a lower mean piglet birth weight in sows fed the high-fiber diet. More recently, a large Danish study with 3,163 sows did not report any impact on total born when comparing a diet high in SBP (450 g SBP/d on average) with normal gestation feed (Sørensen et al., 2016). The observed increase in total born piglets with increased inclusion of fiber in the present study could also be explained by the increased energy intake in early gestation. In line with that, Hoving et al. (2011) found an increased litter size in sows fed additional 30% energy during the first 30 d of gestation as compared with no additional energy or additional 30% energy from dietary protein (Hoving et al., 2011). The present study clearly indicates that high levels of DF have no negative consequences for reproductive output.

### Fiber and protein utilization

The N retention increased with increasing fiber, mainly because N intake increased while the urinary N output decreased. As a consequence, N efficiency for retention increased with inclusion of fiber from 23% to 39% in early gestation. In line with this, Yang et al. (2021) found that N efficiency increased from 41% to 50% when adding a fiber mixture to a low-fiber control diet in early gestation. The altered N metabolism toward less N loss in urine and more loss via feces in response to increasing DF was, apart from lower N digestibility, most likely also due to greater recirculation of urea from the blood to the intestinal lumen (Jarrett and Ashworth, 2018; Yang et al., 2021). In line with this, Yang et al. (2021) found that 60% to 80% of total N in feces was microbial protein N and that the inclusion of fiber increased the amount of microbial protein, indicating a greater population and likely also greater diversity of the microbiota in response to fiber inclusion.

## Conclusion

This study showed that gestating sows efficiently utilize energy from SBP and that increasing levels of DF improved the N retention and N utilization. Concomitantly a numerical improvement in fat retention was observed. This emphasizes the importance of understanding energy utilization within the animal to be able to formulate the dietary composition correctly taking the energy value of feedstuffs into account. In future studies, it is recommended to focus on interactions between DF and FS and between DF and dietary protein sources, as they may affect the nutrient absorption dynamics.

## Abbreviations

ADFI: average daily feed intake
AF: atomic fraction
ATTD: apparent total tract digestibility
BF: backfat
BW: bodyweight
CP: crude protein
DF: dietary fiber
DM: dry matter; D_2_O, deuterium oxide
FS: feeding strategy
GE: gross energy
HE: heat energy
ME: metabolizable energy
NEFA: nonesterified fatty acid; N, nitrogen
NSP: nonstarch polysaccharide
RE: retained energy
RF: retained (body) fat
RFE: retained fat energy
RP: retained (body) protein
RPE: retained protein energy
SBP: sugar beet pulp
SCFA: short-chain fatty acid;
TiO_2_: titanium dioxide

## References

[CIT0001] AOAC. 2000. Official methods of analysis. 17th ed. Gaithersburg (MD): Association of Analytical Chemist.

[CIT0002] Bach Knudsen, K. E. 1997. Carbohydrate and lignin contents of plant materials used in animal feeding. Anim. Feed Sci. Technol. 67:319–338. doi:10.1016/s0377-8401(97)00009-6

[CIT0003] Brouns, F., S. A. Edwards, and P. R. English. 1994. Effect of dietary fibre and feeding system on activity and oral behaviour of group housed gilts. Appl. Anim. Behav. Sci. 39:215–223. doi:10.1016/0168-1591(94)90157-0

[CIT0004] Bruun, T. S., M. Eskildsen, C. K. Hojgaard, N. P. Nørskov, K. E. Bach Knudsen, P. K. Theil, and T. Feyera. 2023. Feeding level during the last week of gestation can influence performance of sows and their litters in the subsequent lactation. J. Anim. Sci 101:1–11. doi:10.1093/jas/skad349 PMC10590173 37813381

[CIT0005] Burkhalter, T. M., N. R. Merchen, L. L. Bauer, S. M. Murray, A. R. Patil, J. L. Brent, Jr., and G. C. Fahey, Jr. 2001. The ratio of insoluble to soluble fiber components in soybean hulls affects ileal and total-tract nutrient digestibilities and fecal characteristics of dogs. J. Nutr. 131:1978–1985. doi:10.1093/jn/131.7.1978 11435517

[CIT0006] Danielsen, V., and E. -M. Vestergaard. 2001. Dietary fibre for pregnant sows: effect on performance and behaviour. Anim. Feed Sci. Technol. 90:71–80. doi:10.1016/s0377-8401(01)00197-3

[CIT0007] Dourmad, J. Y., M. Etienne, A. Valancogne, S. Dubois, J. van Milgen, and J. Noblet. 2008. InraPorc: a model and decision support tool for the nutrition of sows. Anim. Feed Sci. Technol. 143:372–386. doi:10.1016/j.anifeedsci.2007.05.019

[CIT0008] EvaPig. 2008. Evaluation of pigs feed. Reference manual. [accessed April 5, 2021]. http://www.evapig.com/

[CIT0009] Hansen, B. 1989. Determination of nitrogen as elementary N, an alternative to Kjeldahl. Acta Agric. Scand. 39:113–118. doi:10.1080/00015128909438504

[CIT0010] Hoving, L. L., N. M. Soede, C. M. C. van der Peet-Schwering, E. A. M. Graat, H. Feitsma, and B. Kemp. 2011. An increased feed intake during early pregnancy improves sow body weight recovery and increases litter size in young sows. J. Anim. Sci. 89:3542–3550. doi:10.2527/jas.2011-3954 21705632

[CIT0011] Jakobsen, K., P. K. Theil, and H. Jørgensen. 2005. Methodological considerations as to quantify nutrient and energy metabolism in lactating sows. J. Anim. Feed Sci. 14:31–47. doi:10.22358/jafs/70353/2005

[CIT0012] Jarrett, S., and C. J. Ashworth. 2018. The role of dietary fibre in pig production, with a particular emphasis on reproduction. J. Anim. Sci. Biotechnol. 9:59. doi:10.1186/s40104-018-0270-0 30128149 PMC6091159

[CIT0013] Jorgensen, B., and M. T. Sorensen. 1998. Different rearing intensities of gilts: II. Effects on subsequent leg weakness and longevity. Livest. Prod. Sci. 54:167–171. doi:10.1016/s0301-6226(97)00177-2

[CIT0014] Jørgensen, H., P. K. Theil, and K. E. Bach Knudsen. 2011. Enteric methane emission from pigs. In: Carayannis, E., editor. Planet earth 2011 *– global warming challenges and opportunities for policy and practice*. Rijeka (Croatia): InTech; p. 605–623.

[CIT0015] Kil, D. Y., B. G. Kim, and H. H. Stein. 2013. Feed energy evaluation for growing pigs. Asian Australas. J. Anim. Sci. 26:1205–1217. doi:10.5713/ajas.2013.r.02 25049902 PMC4093404

[CIT0016] Krogh, U., T. S. Bruun, J. Poulsen, and P. K. Theil. 2017. Impact of fat source and dietary fibers on feed intake, plasma metabolites, litter gain and the yield and composition of milk in sows. Animal. 11:975–983. doi:10.1017/S1751731116002585 27903321

[CIT0017] Krogh, U., M. Eskildsen, M. T. Sørensen, H. Jørgensen, and P. K. Theil. 2018. Heart rate as predictor of heat production at different reproductive stages in second parity free-ranging sows. In: Roura, E. and F. Dunshea, editors, Proceedings of the 14th International Symposium on Digestive Physiology of Pigs, 21st to 24th of August, 2018. Cambridge (UK): Cambridge University Press; p. 491.

[CIT0018] Maes, D. G. D., G. P. J. Janssens, P. Delputte, A. Lammertyn, and A. de Kruif. 2004. Back fat measurements in sows from three commercial pig herds: relationship with reproductive efficiency and correlation with visual body condition scores. Livest. Prod. Sci. 91:57–67. doi:10.1016/j.livprodsci.2004.06.015

[CIT0019] Olesen, C. S., H. Jørgensen, and V. Danielsen. 2001. Effect of dietary fibre on digestibility and energy metabolism in pregnant sows. Acta Agric. Scand. A Anim. Sci. 51:200–207. doi:10.1080/090647001750367294

[CIT0020] Patience, J. F. 2012. Feed efficiency in swine. 1st rev. ed. Wageningen (The Netherlands): Wagening Acedemic Press.

[CIT0021] Pedersen, T. F. 2020. High litter size with high piglet survival. – [accessed March 16, 2022]. https://danbred.com/high-litter-size-with-high-piglet-survival/

[CIT0023] Peltoniemi, O., S. Bjorkman, and D. Maes. 2016. Reproduction of group-housed sows. Porcine Health Manag. 2:2–15. doi:10.1186/s40813-016-0033-2 28405441 PMC5382509

[CIT0024] Peltoniemi, O., J. Yun, S. Bjorkman, and T. Han. 2021. Coping with large litters: the management of neonatal piglets and sow reproduction. J. Anim. Sci. Technol. 63:1–15. doi:10.5187/jast.2021.e3 33987579 PMC7882835

[CIT0025] Priester, M., C. Visscher, M. Fels, K. Rohn, and G. Dusel. 2020. Fibre supply for breeding sows and its effects on social behaviour in group-housed sows and performance during lactation. Porcine Health Manag. 6:15. doi:10.1186/s40813-020-00153-3 32518669 PMC7273647

[CIT0026] Prunier, A., M. Heinonen, and H. Quesnel. 2010. High physiological demands in intensively raised pigs: impact on health and welfare. Animal. 4:886–898. doi:10.1017/S175173111000008X 22444261

[CIT0027] Ramonet, Y., J. van Milgen, J. -Y. Dourmad, S. Dubois, M. Meunier-Salaun, and J. Noblet. 2000. The effect of dietary fibre on energy utilisation and partitioning of heat production over pregnancy in sows. Br. J. Nutr. 84:85–94. doi:10.1017/S0007114500001264. 10961164

[CIT0028] Ren, P., X. J. Yang, J. S. Kim, D. Menon, D. Pangeni, H. Manu, A. Tekeste, and S. K. Baidoo. 2017. Plasma acyl ghrelin and nonesterified fatty acids are the best predictors for hunger status in pregnant gilts. J. Anim. Sci. 95:5485–5496. doi:10.2527/jas2017.1785 29293797 PMC6292324

[CIT0029] Renteria-Flores, J. A., L. J. Johnston, G. C. Shurson, and D. D. Gallaher. 2008. Effect of soluble and insoluble fiber on energy digestibility, nitrogen retention, and fiber digestibility of diets fed to gestating sows. J. Anim. Sci. 86:2568–2575. doi:10.2527/jas.2007-0375 18539846

[CIT0030] Rijnen, M., M. W. A. Verstegen, M. J. W. Heetkamp, J. Haaksma, and J. W. Schrama. 2001. Effects of dietary fermentable carbohydrates on energy metabolism in group-housed sows. J. Anim. Sci. 79:148–154. doi:10.2527/2001.791148x. 11204695

[CIT0031] Rijnen, M., M. W. A. Verstegen, M. J. W. Heetkamp, J. Haaksma, and J. W. Schrama. 2003. Effects of dietary fermentable carbohydrates on behavior and heat production in group-housed sows. J. Anim. Sci. 81:182–190. doi:10.2527/2003.811182x 12597389

[CIT0032] Rozeboom, D. W., J. E. Pettigrew, R. L. Moser, S. G. Cornelius, and S. M. Elkandelgy. 1994. In-vivo estimation of body composition of mature gilts using live weight, backfat thickness, and deuterium oxide. J. Anim. Sci. 72:355–366. doi:10.2527/1994.722355x 8157520

[CIT0033] SEGES. 2019. H9A – Body condition and feeding strategies (Huld og foderkurver): Repromanagement Manual. Aarhus N (Denmark): SEGES Danish Pig Research Centre.

[CIT0034] Serena, A., H. Jorgensen, and K. E. B. Knudsen. 2009. Absorption of carbohydrate-derived nutrients in sows as influenced by types and contents of dietary fiber. J. Anim. Sci. 87:136–147. doi:10.2527/jas.2007-0714 18676728

[CIT0035] Short, F. J., P. Gorton, J. Wiseman, and K. N. Boorman. 1996. Determination of titanium dioxide added as an inert marker in chicken digestibility studies. Anim. Feed Sci. Technol. 59:215–221. doi:10.1016/0377-8401(95)00916-7

[CIT0036] Sørensen, G., L. U. Hansen, and M. F. Nielsen. 2016. Sugarbeet pulp as supplement to feed to gestating animals [Roepiller som supplement til foder til drægtige dyr]. SEGES report no. 1076. Aarhus N (Denmark). Danish. [accessed March 16, 2022]. https://svineproduktion.dk/publikationer/kilder/lu_medd/2016/1076

[CIT0037] Strathe, A. V., T. S. Bruun, and C. F. Hansen. 2017. Sows with high milk production had both a high feed intake and high body mobilization. Animal. 11:1913–1921. doi:10.1017/S1751731117000155 28196552

[CIT0039] Theil, P. K., H. Jørgensen, and K. Jakobsen. 2002a. Energy and protein metabolism in pregnant sows fed two levels of dietary protein. J. Anim. Physiol. Anim. Nutr. (Berl). 86:399–413. doi:10.1046/j.1439-0396.2002.00404.x 12534833

[CIT0041] Theil, P. K., T. T. Nielsen, N. B. Kristensen, R. Labouriau, V. Danielsen, C. Lauridsen, and K. Jakobsen. 2002b. Estimation of milk production in lactating sows by determination of deuterated water turnover in three piglets per litter. Acta Agric. Scand. A Anim. Sci. 52:221–232. doi:10.1080/090647002762381104

[CIT0040] Theil, P. K., H. Jørgensen, and K. Jakobsen. 2004. Energy and protein metabolism in lactating sows fed two levels of dietary fat. Livest. Prod. Sci. 89:265–276. doi:10.1016/j.livprodsci.2004.01.001

[CIT0038] Theil, P., A. Chwalibog, and H. Jørgensen. 2020. Energy for pigs: metabolism, requirement, utilisation and prediction of dietary content. In: Bach Knudsen, K. E., N. J. Kjeldsen, H. D. Poulsen and B. B. Jensen, editors. Nutritional physiology of pigs. Chapter 20, pp. 1–106. [accessed March 16, 2022]. https://svineproduktion.dk/Services/Undervisningsmateriale2

[CIT0043] Tybirk, P., A.B. Strathe, E. Vils, N.M. Sloth and S. Boisen. 2006. Det danske Fodervurderingssystem til svinefoder. Report no. 36, Dansk Svineproduktion. [accessed January 5, 2024]. https://svineproduktion.dk/publikationer/kilder/lu_rapporter/30/.

[CIT0042] Tybirk, P., N. M. Sloth, N. Kjeldsen, and N. Weber. 2020. Danish nutrient standards. 30th ed. Aarhus N (Denmark): Seges Danish Pig Research Centre.

[CIT0044] van der Peet-Schwering, C. M. C., B. Kemp, G. P. Binnendijk, L. A. den Hartog, H. A. M. Spoolder, and M. W. A. Verstegen. 2003. Performance of sows fed high levels of nonstarch polysaccharides during gestation and lactation over three parities. J. Anim. Sci. 81:2247–2258. doi:10.2527/2003.8192247x 12968700

[CIT0045] Yang, M., Z. Y. Mao, X. M. Jiang, P. Cozannet, L. Q. Che, S. Y. Xu, Y. Lin, Z. F. Fang, B. Feng, J. P. Wang, et al. 2021. Dietary fiber in a low-protein diet during gestation affects nitrogen excretion in primiparous gilts, with possible influences from the gut microbiota. J. Anim. Sci. 99:14. doi:10.1093/jas/skab121 PMC8174468 33871635

[CIT0046] Young, M. G., M. D. Tokach, F. X. Aherne, R. G. Main, S. S. Dritz, R. D. Goodband, and J. L. Nelssen. 2004. Comparison of three methods of feeding sows in gestation and the subsequent effects on lactation performance. J. Anim. Sci. 82:3058–3070. doi:10.2527/2004.82103058x 15484959

[CIT0047] Zhou, P., P. K. Theil, D. Wu, and K. E. B. Knudsen. 2018. In vitro digestion methods to characterize the physicochemical properties of diets varying in dietary fibre source and content. Anim. Feed Sci. Technol. 235:87–96. doi:10.1016/j.anifeedsci.2017.11.012

[CIT0048] Zhou, Y. F., X. M. Zhang, C. Wang, H. K. Wei, S. W. Jiang, and J. Peng. 2019. Effects of North American and Danish feeding strategies on the reproductive performance of American Landrace-Yorkshire crossbred sows during gestation. Livest. Sci. 228:67–71. doi:10.1016/j.livsci.2019.07.025

